# Cosmesis and Body Image After Robotic Sacrocolpopexy

**DOI:** 10.1007/s00192-026-06615-6

**Published:** 2026-04-01

**Authors:** Sarah Ashmore, Kimberly Kenton, Alesha Kotian, Keilah Dos Santos, Anna Hornbach, Kelsey Nichols, Margaret G. Mueller

**Affiliations:** 1 Section of Urogynecology and Reconstructive Pelvic Surgery, University of Chicago, Chicago, IL USA; 2 Department of Obstetrics and Gynecology, University of Chicago, Chicago, IL USA; 3 Michigan State University College of Human Medicine, Grand Rapids, MI USA

**Keywords:** Body image, Cosmesis, Robotic sacrocolpopexy, Single port sacrocolpopexy, Patient satisfaction, Surgical scars

## Abstract

**Introduction and Hypothesis:**

Satisfaction of surgical scars after robotic sacrocolpopexy have not yet been explored.

**Objective:**

To determine differences in patient perception of body image and cosmesis following single port (SP) versus traditional multiport (TMP) robotic sacrocolpopexy.

**Methods:**

We performed a prospective cohort study at a single tertiary care center of women undergoing robotic sacrocolpopexy between September 2023 and December 2024. Women undergoing SP or TMP robotic sacrocolpopexy for prolapse were included. At 3 months after surgery, participants completed the Body Image Questionnaire (BIQ). The BIQ is a 10-item survey which includes a body image, cosmetic, and self-confidence scale used to assess a patient’s perception of body image and cosmesis after surgery. Low scores on the body image scale and high scores on the cosmetic indicated higher patient satisfaction. Changes in preoperative and postoperative self-confidence scores were calculated. A positive change in scores was associated with higher self-confidence after surgery.

**Results:**

One hundred fourteen participants underwent robotic sacrocolpopexy: 63 (45%) SP and 77 (55%) TMP robot. Median (range) body image scale and cosmetic scale scores for the entire cohort were 5 (5–16) and 20 (5–24), respectively. Median preoperative and postoperative self-confidence scores were 8 (0–10) and 8 (2–10), respectively. There were no significant differences in total body image scale (*p* = 0.55) and cosmetic scale (*p* = 0.17) scores between groups. Change in self-confidence score after surgery was also similar (*p* = 0.14).

**Conclusions:**

Women were highly satisfied with their body image and cosmesis of surgical scars after robotic sacrocolpopexy without significant differences between SP and TMP approaches.

## Introduction

For a patient, incisional scars are often the only visible evidence of a prior surgery. Cosmesis of surgical scars and scar prevention or reduction strategies are highly valued by patients [1]. Scarring can negatively impact a patient’s quality of life and decrease satisfaction after elective surgery [1–3]. Often, surgeons and patients’ perception of surgical scars do not align [4]. A surgeon may fail to recognize what characteristics of a scar are most important to a patient [1, 5].

Single site (SS) laparoscopy offers improved postoperative pain and cosmesis compared to traditional multiport (TMP) laparoscopy and open techniques [6–10]. Minimally invasive surgery continues to advance with the evolution of SS and single port (SP) robotic surgery. The SS platform introduces multiple robotic arms through a single robotic port, which can result in instrument clashing and decreased range of motion [11]. The SP robot overcomes the challenges of the SS platform with a single robotic arm which introduces three instruments and a camera through a single multichannel port. Literature surrounding the SP robotic platform within gynecology is limited and studies comparing outcomes to TMP robotic surgery are sparse. Despite this, SP robotic surgery is gaining popularity within gynecology.

Cosmetic outcomes and patient satisfaction with body image following SP robotic surgery has not yet been explored. We aim to assess if patient satisfaction with body image and cosmesis of surgical scars are different after SP and TMP robotic sacrocolpopexy. We hypothesize that women will have higher satisfaction with a single umbilical incision compared to multiple small incisions across the abdomen.

## Study Design

**Fig. 1:**
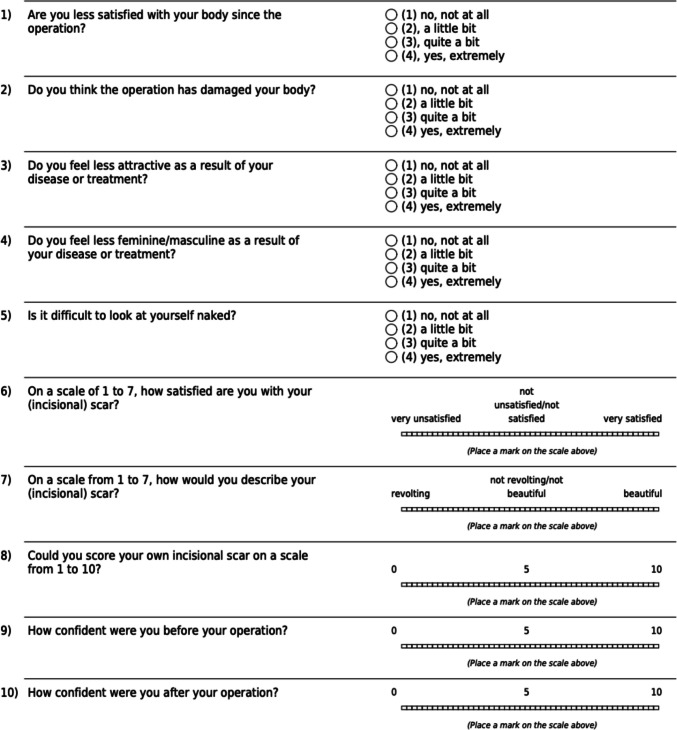
Body Image Questionnaire

We performed a prospective cohort study of women undergoing robotic sacrocolpopexy at a single tertiary care center. Institutional review board approval (IRB24-0113) was obtained prior to study enrollment. Surgeries were performed by six board-certified Urogynecology and Reconstructive Pelvic Surgeons between September 2023 and December 2024. Women scheduled for SP or TMP robotic sacrocolpopexy for uterovaginal or vaginal vault prolapse were approached prior to surgery. The use of the SP or TMP robotic platform was at the discretion of the surgeon. Any concomitant minimally invasive prolapse or incontinence procedures were allowed. Women were excluded if they were non-English speaking or unable to complete study questionnaires. Written consent was obtained prior to study enrollment. The primary objective was to assess differences in patient perception of body image and cosmesis of surgical scars after SP versus TMP robotic sacrocolpopexy. The Body Image Questionnaire (BIQ) was used as the primary outcome (Fig. 1). Differences in responses to individual BIQ items and the Patient Global Impression of Improvement (PGI-I) were analyzed as secondary objectives.

The BIQ is a 10-item validated survey evaluating a patient’s perception of their body image, cosmesis, and self-confidence after surgery [12]. The BIQ can be divided into a body image scale (questions 1–5), a cosmesis scale (questions 6–8), and a self-confidence scale (questions 9 and 10). The body image scale measures perception and satisfaction of bodily appearance. Answers are calculated on a scale of 5 to 20 with lower scores indicating higher patient satisfaction. The cosmetic scale assesses satisfaction with the physical appearance of surgical scars. Answers are calculated on a scale of 3 to 24 with higher scores indicating higher patient satisfaction. The self-confidence scale explores patient’s self-confidence before and after surgery. Both questions are scored on a scale of 0–10 with higher scores indicating greater levels of self-confidence.

**Fig. 2:**
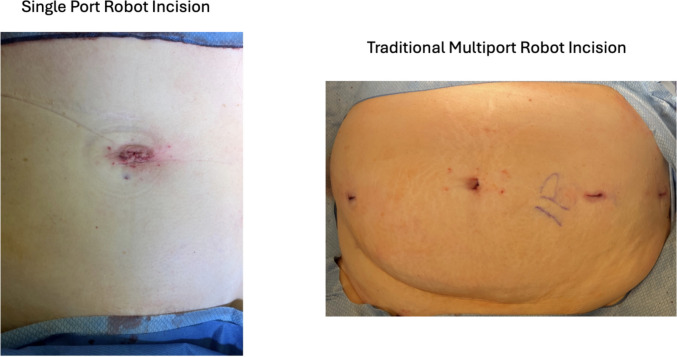
Differences in surgical incisions for single port versus traditional multiport robotic sacrocolpopexy

During SP robotic cases, a 15–20 mm infraumbilical skin incision was made and abdominal access was obtained through an open Hassan technique. The fascial incision was extended between 20 and 25 mm. No additional accessory ports were placed. During TMP robotic cases, the abdominal entry was dependent on surgeon preference. Four surgeons entered with a Veress needle at the base of the umbilicus followed by placement of the first robotic trocar under direct visualization. Two surgeons entered via an open Hassan technique at the base of the umbilicus through a 10 mm skin incision. Two robotic trocars were next placed on the patient’s left side and one of the right side. One surgeon routinely placed an 8 mm assistant port in the right upper quadrant. Figure 2 displays the differences in surgical incisions made at the time of SP and TMP robotic sacrocolpopexy.

Demographics, clinical characteristics, intraoperative and postoperative outcomes were abstracted from the medical chart through 3 months after surgery. Preoperative prolapse severity was determined using the pelvic organ prolapse quantification scale. As part of routine care, women received a telephone call on postoperative day one (POD1) from our nursing team. Participants were next seen via telemedicine at 2-weeks unless an in person visit was deemed necessary. At 3 months after surgery, an in person or telemedicine visit was completed. A pelvic exam was only completed at the time of an in person visit. At each visit, women are asked to rate their pain on a 10-point numeric rating scale and vaginal bulge symptoms were assessed. Recurrent prolapse was defined as evidence of prolapse beyond the hymen, any new bothersome vaginal bulge symptoms, or any retreatment for prolapse. The BIQ and PGI-I were distributed at the 3-month visit.

Statistical analysis was performed using IBM SPSS Statistics version 29 (SPSS Inc, Armonk, NY). Standard group comparisons were performed. Chi square Fisher’s exact test was used for categorical variables. For continuous variables, independent student’s *t*-test was used for normally distributed data and Mann–Whitney *U* test for not normally distributed data.

## Results

One hundred seventy-seven women were approached and 140 consented to participate in our study. Sixty-three (45%) robotic sacrocolpopexies were performed on the SP platform and 77 (55%) on the TMP platform (Table 1). Median (range) age of the entire cohort was 64 years (36–86) and body mass index (BMI) was 26.3 mg/kg^2^ (17.0–46.1). The majority of women were White (63.6%), non-Hispanic (60.7%), had private insurance (57.9%), and were postmenopausal (79.3%). Advanced pelvic organ prolapse (stage III or IV) was most common (71.4%). Concomitant hysterectomy and synthetic miduretheral sling were performed in 37 (26.4%) and 102 (72.9%) surgeries, respectively. Median estimated blood loss was 50 mL (0–300) and operative time was 239 min (95–561). One (0.7%) intraoperative complication occurred during the study. A cystotomy occurred during the anterior dissection which was recognized immediately and repaired in standard fashion. Median postoperative pain score on day 1 and day 14 were 3 (0–10) and 0 (0–8), respectively. At 3-months after surgery, 72.8% had stage 0 or I prolapse on exam. Two participants (1.4%) described a new bothersome vaginal bulge with stage II or greater prolapse on exam. One (0.7%) elected for repeat reconstructive pelvic surgery. Twelve (8.6%) women experienced a postoperative complication within 3 months of surgery: three (2.1%) ileus, one (0.7%) incisional hernia, two (1.4%) vaginal mesh/suture erosion, four (2.8%) voiding dysfunction > 7 days postoperative, and two (1.4%) superficial surgical site infections. Readmission rate was 2.1% and reoperation rate was 3.6%. All readmissions were for conservative management of postoperative ileus. Reoperations included: vaginal mesh excision (3.6%), incisional hernia repair (0.7%), additional prolapse surgery (0.7%), and additional incontinence surgery (0.7%). There were no significant difference between the SP and TMP cohorts outside of age and operative time. Women in the SP cohort were younger (61 years versus 65, *p* = 0.05) and surgeries performed on the SP platform were 41 min shorter (221 min versus 262, *p* = 0.003).

**Table 1:** Demographics, clinical characteristics, and surgical outcomes

Variable	Entire cohort (*n* = 140)	SP (*n* = 63)	TMP (*n* = 77)	*p*
Age; *years*	64 (36–86)	61 (36–78)	65 (36–86)	0.05
BMI; *mg/kg*^*2*^	26.3 (17.0–46.1)	24.6 (18.4–46.1)	26.9 (17.0–44.2)	0.10
Ethnicity				
Hispanic or Latino Not Hispanic or Latino	5 (3.6%) 85 (60.7%)	1 (1.6%) 40 (63.5%)	4 (5.2%) 45 (58.4%)	0.49
Race				
White Black or African American Asian or Indian	89 (63.6%) 6 (4.3%) 2 (1.4%)			
		42 (66.7%) 2 (3.2%) 0 (0.0%)	47 (61.0%) 4 (5.2%) 2 (2.6%)	0.54
Insurance				
Private Medicare/Medicaid	81 (57.9%) 59 (42.1%)	40 (63.5%) 23 (36.5%)	41 (53.2%) 36 (46.8%)	0.23
Postmenopausal; *yes*	111 (79.3%)	48 (76.2%)	63 (81.8%)	0.53
Preoperative POPQ				1.00
Stage II Stage III Stage IV	40 (28.6%) 94 (67.1%) 6 (4.3%)	12 (19.0%) 47 (74.6%) 5 (6.4%)	28 (36.4%) 47 (61.0%) 2 (2.6%)	
Concomitant hysterectomy; *yes*	37 (26.4%)	14 (22.2%)	23 (29.9%)	0.64
Concomitant midurethral sling; *yes*	102 (72.9%)	29 (46.0%)	53 (68.8%)	0.20
Estimated blood loss; *mL*	50 (0–300)	50 (10–300)	50 (0–200)	0.21
Operative time; *minutes*	239 (95–561)	221 (155–414)	262 (95–561)	0.003
Intraoperative complications; *yes*	1 (0.7%)	0 (0.0%)	1 (1.3%)	1.00
POD1 Pain Score	3 (0–10)	3 (0–8)	3 (0–10)	0.26
POD14 Pain Score	0 (0–8)	0 (0–5)	0 (0–8)	0.95
3-month POPQ				0.33
Stage 0 Stage I Stage II Stage III Telemedicine Unknown	79 (56.4%) 23 (16.4%) 6 (4.3%) 1 (0.7%) 8 (5.7%) 23 (16.4%)	38 (60.3%) 10 (15.9%) 4 (6.3%) 0 (0.0%) 1 (1.6%) 10 (15.9%)	41 (53.2%) 13 (16.9%) 2 (2.6%) 1 (1.3%) 7 (9.1%) 13 (16.9%)	
3-month vaginal bulge symptoms; *yes*	2 (1.4%)	1 (1.6%)	1 (1.3%)	0.99
Recurrent prolapse;* yes*	2 (1.4%)	1 (1.6%)	1 (1.3%)	0.99
Postoperative complication Ileus Incisional hernia Vaginal mesh/suture erosion Voiding dysfunction > 7 days postoperative Superficial surgical site infection	12 (8.6%) 3 (2.1%) 1 (0.7%) 2 (1.4%) 4 (2.8%) 2 (1.4%)	5 (7.9%) 1 (1.6%) 0 (0.0%) 1 (1.6%) 2 (3.2%) 1 (1.6%)	7 (9.1%) 2 (2.6%) 1 (1.3%) 1 (1.3%) 2 (2.6%) 1 (1.3%)	0.81
Readmission rate	3 (2.1%)	1 (1.6%)	2 (2.6%)	0.68
Reoperation rate Vaginal mesh excision Incisional hernia repair Prolapse surgery Incontinence surgery	5 (3.6%) 2 (1.4%) 1 (0.7%) 1 (0.7%) 1 (0.7%)	2 (3.2%) 1 (1.6%) 0 (0.0%) 0 (0.0%) 1 (1.6%)	3 (3.4%) 1 (1.3%) 1 (1.3%) 1 (1.3%) 0 (0.0%)	1.00

All participants completed the BIQ and PGI-I at 3 months after surgery (Table 2). For the body image scale, median total score for the entire cohort was 5 (5–16). Total body image scale scores did not differ between the SP and TMP cohorts (5 versus 5, *p* = 0.55). When analyzing each component of the body image scale, answers were similar between robotic platforms. The median total score for the cosmetic scale was 20 (5–24) for the entire cohort. Median scores were 20 (9–24) for the SP cohort and 20 (5–24) for the TMP cohort (*p* = 0.17). Individual answers for each question of the cosmetic scale were similar between groups. Median preoperative self-confidence score was 8 (0–10) for the entire cohort. Median postoperative self-confidence score was 8 (2–10); therefore, there was no change in self-confidence scores for the entire cohort after surgery. Self-confidence scores before (*p* = 0.29) and after (*p* = 0.91) surgery were similar between groups. Change in self-confidence scores before and after surgery were also similar between groups (*p* = 0.14). On PGI-I answers, 65.0% reported they were “very much better,” 21.4% were “much better,” and 6.4% were “a little better” at 3 months after surgery (Table 2). PGI-I answers were not significantly different between groups (*p* = 0.86).

**Table 2:** Body Image Questionnaire and Patient Global Impression of Improvement outcomes

Patient reported outcomes	Entire cohort (*n* = 140)	SP (*n* = 63)	TMP (*n* = 77)	*p*
Body image scale; *total score*	5 (5–16)	5 (5–16)	5 (5–13)	0.55
Question 1 Question 2 Question 3 Question 4 Question 5	1 (1–4) 1 (1–3) 1 (1–4) 1 (1–4) 1 (1–3)	1 (1–4) 1 (1–3) 1 (1–4) 1 (1–4) 1 (1–4)	1 (1–4) 1 (1–3) 1 (1–4) 1 (1–4) 1 (1–4)	0.35 0.43 0.39 0.12 0.55
Cosmetic scale; *total score* Question 6 Question 7 Question 8	20 (5–24) 7 (1–7) 5 (3–10) 8.5 (3–10)	20 (9–24) 7 (2–7) 6 (3–7) 9 (4–10)	20 (5–24) 7 (1–7) 5 (2–7) 8 (3–10)	0.17 0.34 0.22 0.33
Self-confidence scale				
Preoperative Postoperative Change in score	8 (0–10) 8 (2–10) 0 (−8–10)	8 (0–10) 8 (2–10) 0 (−8–10)	8 (0–10) 8 (2–10) 0 (−8–10)	0.29 0.91 0.14
PGI-I				0.86
Very much better Much better A little better No change A little worse Much worse Very much worse	91 (65.0%) 30 (21.4%) 9 (6.4%) 3 (2.1%) 2 (1.4%) 4 (2.9%) 1 (0.7%)	43 (68.3%) 12 (19.0%) 5 (7.9%) 1 (1.6%) 1 (1.6%) 1 (1.6%) 0 (0.0%)	48 (62.3%) 18 (23.4%) 4 (5.2%) 2 (2.6%) 1 (2.3%) 3 (3.9%) 1 (1.3%)	

## Discussion

Women were highly satisfied with the cosmesis of their surgical scars and body image after both SP and TMP robotic sacrocolpopexy. Self-confidence scores were high before and after surgery without any significant change in scores. There were no significant differences in patient perception of body image, cosmesis of surgical scars, or self-confidence between SP and TMP robotic sacrocolpopexy. Overall, women were pleased with robotic sacrocolpopexy outcomes.

Minimally invasive surgery is superior to open techniques with decreased perioperative complications and improved recovery [13, 14]. SP surgery offers comparable outcomes to TMP approach with the benefit of a single incision [6, 7, 15]. A single trocar placed at the umbilicus likely results in less trauma compared to the placement of multiple ports across the abdomen that transverse the rectus muscle. Previous studies have reported postoperative pain and cosmetic advantages with SS surgery across other surgical specialties [6–10]. However, the cosmetic impact of SS surgery in gynecology is less understood. Yeung et al. performed a prospective observational study to estimate patient preferences regarding the cosmesis of abdominal incisions used at the time of hysterectomy [16]. The SS incision was the most preferred choice compared to other minimally invasive and open surgical incisions. Song et al. analyzed cosmetic satisfaction after SS gynecologic laparoscopic surgery [17]. Women were asked to complete the BIQ postoperatively. Patient satisfaction was high on both the body image and cosmetic scales. Close to 70% reported the scar was invisible and 97% would recommend SS laparoscopic surgery to others. In a meta-analysis of randomized controlled trials comparing SS and TMP laparoscopic gynecologic surgery, cosmetic outcomes were unchanged between surgical approaches. However, analysis was limited to four trials and three studies utilized a visual analogue scale instead of a validated patient reported outcome measure to assess cosmesis. To the best of our knowledge, we performed one of the first studies to compare cosmetic outcomes of SS versus TMP minimally invasive gynecologic surgery using a validated tool. Similar to prior studies, participants were highly satisfied with the cosmetic outcome of surgical scars without a difference between surgical approaches.

The BIQ assesses a patient’s perception of body image and cosmesis of surgical scars and has been found to have high internal consistency [12]. The BIQ incorporates a series of questions to allow the patient to subjectively analyze their surgical scars. The patient is not provided background information regarding the cosmetic result of alternative surgical approaches. Other authors have added a photo-series questionnaire when distributing the BIQ to determine if the knowledge of alternative surgical cosmetic outcomes impacts a patient’s satisfaction with SS laparoscopic scars [8]. When patients were shown photos of cosmetic outcomes from alternative surgical approaches (i.e., TMP laparoscopy or open), SS scar satisfaction remained significantly higher. This findings highlights an important limitation of our study which may have impacted outcomes. Participants were not provided a photo of the cosmetic result from the alternative surgical approach; therefore, they may not have had a reference for comparison. BIQ scores were high in both the SP and TMP cohorts without a significant difference between scores. More research is needed to analyze if the incorporation of a photo-series would impact patient satisfaction with cosmetic outcomes from SP and TMP robotic surgery.

Pelvic organ prolapse negatively impacts body image and quality of life. In a case–control study, women with prolapse reported decreased body image and overall quality of life compared to women without prolapse [18]. Women with advanced prolapse were also more likely to feel self-conscious about their body, less physically attractive, less feminine, and feel less sexually attractive. In our study, participants were asked to rate their self-confidence on a 10-point scale before and after surgery. Median preoperative self-confidence scales were high for both study groups and there was no significant change after surgery despite 70% having advance prolapse on baseline exam. These findings are inconsistent with prior literature regarding the impact of prolapse on self-confidence. Outcomes from our study could potentially be a result of the timing of self-confidence assessment. Participants were administered the BIQ only at 3 months after surgery. Therefore, they were asked to rate preoperative and postoperative self-confidence 3-months after surgery which introduces the potential for recall bias. The high level of baseline self-confidence could have impacted both the body image and cosmetic scale outcomes.

Our study has several strengths and limitations. Literature surrounding SP robotic gynecologic surgery is limited to a few case series and small comparative studies. SP robotic sacrocolpopexy is a feasible, safe alternative however more research is needed comparing outcomes of SP and TMP robotic platforms [19]. We performed one of the first studies to compare outcomes following SP and TMP robotic sacrocolpopexy. The cosmetic advantage of the SP robotic platform had not been explored previously within gynecology. Our study was performed at a large academic center with six board-certified Urogynecology and Reconstructive Pelvic Surgeons. We are limited by the inherent limitations of a cohort study as we were unable to control for potential variables that could have impacted results. Women were offered either telemedicine or in person postoperative appointments; therefore, not everyone had a documented pelvic exam at 3-months after surgery. Perioperative outcomes such as postoperative complications, readmissions, and reoperations were collected; however, we were limited to our own electronic medical record. If a patient presented to an outside hospital, we were unable to collect these outcomes. The decision to perform the surgery on an SP or TMP robotic platform was left to the discretion of the surgeon and based on surgeon comfort with the different available robotic platforms. If women were candidates for either the SP or TMP robotic platform, the decision was made through a shared decision between the patient and surgeon. Therefore, we could not control for a potential selection bias between the two groups. Differences in surgeon comfort with the different robotic platforms may also impact our perioperative outcomes such as operative time. Our study also purposefully did not include a surgeon assessment of surgical scars and focused only on patient perception. Future research will assess surgeon perception of incisional scars between robotic platforms.

Perception of body image and cosmesis of surgical scars were similar between SP and TMP robotic sacrocolpopexy. Women were highly satisfied overall without differences between surgical approach. The SP robot is a safe, feasible alternative to TMP robotic surgery for women who desire a single umbilical incision. Further research is needed assessing long-term outcomes and potential benefits of operating through a single abdominal incision.

## Data Availability

Data is available upon request.

## Declarations

### Presentation Information

Full Oral Presentation, 2025 AUGS PFD Week 2025, Vancover, Canada, October 2025.
